# Reviewer acknowledgment 2015

**DOI:** 10.1186/s12947-016-0052-9

**Published:** 2016-02-19

**Authors:** Eugenio Picano, Rosa Sicari

**Affiliations:** CNR Institute of Clinical Physiology, Pisa, Italy

## Abstract

The Editors of *Cardiovascular Ultrasound* would like to thank all of our reviewers who have contributed to the journal in Volume 13 (2015) and whose valuable support is fundamental to its success.

**Eustachio Agricola**

Italy

**Lamia Air-Ali**

Italy

**Francesco Ancona**

Italy

**Ana Barac**

USA

**Jutta Bergler-klein**

Austria

**Elisabetta Bianchini**

Italy

**Tonino Bombardini**

Italy

**Adrian Borges**

Germany

**Rosa Maria Bruno**

Italy

**Thomas Buck**

Germany

**Enrico Caiani**

Italy

**Matteo Cameli**

Italy

**David Canty**

Australia

**Romain Capoulade**

USA

**Juan Carlos del Alamo**

USA

**Quirino Ciampi**

Italy

**Victoria Delgado**

Netherlands

**Johan De Sutter**

Belgium

**Pavel Dimitrow**

Poland

**Giovanni Di Salvo**

Saudi Arabia

**Erwan Donal**

France

**Jan Engvall**

Sweden

**Arturo Evangelista**

Spain

**Lothar Faber**

Germany

**Pompilio Faggiano**

Italy

**Francesco Faita**

Italy

**Silvia Favilli**

Italy

**Ricardo Fonseca**

Australia

**Nicola Gaibazzi**

Italy

**Maurizio Galderisi**

Italy

**Luna Gargani**

Italy

**Fabio Guarracino**

Italy

**Hideaki Kanzaki**

Japan

**Jaroslaw Kasprzak**

Poland

**Fabian Knebel**

Germany

**Patrizio Lancellotti**

Belgium

**Davide Lazzeroni**

Italy

**Vincenzo Lionetti**

Italy

**Piotr Lipiec**

Poland

**André Lourenco**

Portugal

**Jorge Lowenstein**

Argentina

**Julien Magne**

France

**Marco Magnoni**

Italy

**Dekleva Milica**

Serbia

**Antonella Moreo**

Italy

**Aleksandar Neskovic**

Serbia

**Stefano Nistri**

Italy

**Gaetano Nucifora**

Italy

**Daniele Panetta**

Italy

**Gianni Pedrizzetti**

Italy

**Mauro Pepi**

Italy

**Bruno Pinamonti**

Italy

**Bogdan Alexandru Popescu**

Romania

**Thomas Porter**

USA

**Lorenza Pratali**

Italy

**Eva Prescott**

Denmark

**Susanna Price**

UK

**Fausto Rigo**

Italy

**Monica Rosca**

Romania

**Antti Saraste**

Finland

**Sebastian Sarvari**

Norway

**Mario Sénéchal**

Canada

**Roxy Senior**

UK

**Divya Shakti**

USA

**John Simpson**

UK

**Massimo Slavich**

Italy

**Ivan Stankovic**

Serbia

**Francesco Stea**

Italy

**Giovanni Tripepi**

Italy

**Albert Varga**

Hungary

**Lucia Venneri**

UK

**Jens-Uwe Voigt**

Belgium

**Giovanni Volpicelli**

Italy

**Xin Zeng**

USA

